# Trauma-Induced Rupture of Liver Hydatid Cyst: A Rare Cause of Anaphylactic Shock

**DOI:** 10.1055/s-0041-1740624

**Published:** 2021-12-23

**Authors:** Charif Khaled, Antoine Kachi

**Affiliations:** 1 Department of General Surgery, Faculty of Medical Sciences, Lebanese University, Beirut, Lebanon; 2 Department of General Surgery, University Medical Center, Lebanese Hospital Geitaoui, Beirut, Lebanon

**Keywords:** hydatid disease, hydatid cyst, hydatid cyst rupture, anaphylactic shock

## Abstract

Hydatid disease is rare; nevertheless, several areas of the world are endemic. Lebanon is one of the endemic countries. This disease requires careful management, as its diagnosis is tough, and its complications are severe and can lead to sudden death. These complications include fistulas, infection, and rupture. Rupture of a hydatid cyst can mimic acute abdomen and show an array of nonspecific symptoms. It could be mistaken for hemorrhagic shock, trauma, or injury to an intra-abdominal organ. The diagnosis of ruptured hydatid cyst should be kept in mind in cattle-raising countries. We report the case of a polytrauma patient who was suspected to have severe intra-abdominal bleeding and hemorrhagic shock, but imaging and laparotomy showed the rupture of a liver hydatid cyst that drove the patient into anaphylactic shock. This article reviews similar cases in the literature and discusses the diagnostic tools, appropriate management, and expected complications.

Hydatid disease or echinococcosis is a zoonotic infection endemic in the Mediterranean, Middle East, North Africa, South America, New Zealand, and Australia. Incidence is around 1–200/100,000. 1 Humans are accidental intermediate hosts. They contract the disease after the ingestion of foods (mainly vegetables) contaminated with embryonated eggs found in infected dog feces. 1 The parasite mainly finds its way to the liver, lungs and brain, 2 and less commonly to the spleen, kidneys, heart, bones, eyes and breast. This parasite forms hydatid cysts that generally go unnoticed until they reach a considerable size. Complications are caused by compressive signs/symptoms, fistulae, bacterial infection, and less commonly intraperitoneal or intrabiliary rupture. 3 All can be fatal if not diagnosed and managed in time.

## Case Presentation

This is the case of a 22-year-old male Syrian construction worker who presented to the emergency department post 4 m fall. He was awake and oriented, with no mention of loss of consciousness. He described landing face down and on the right side of his body. His vitals upon presentation showed tachycardia at 110 beats per minute (BPM) and hyperthermia at 38 °C. A complete neuro examination was normal. Cardiopulmonary auscultation was also normal. He had tenderness over his right lower ribs. His abdomen was soft but with diffuse tenderness on deep palpation, especially in the right upper quadrant. Musculoskeletal examination showed only mild bruising.

Initial management included two large-bore intravenous (IV) lines, blood typing, complete blood count (CBC), coagulation panel, creatinine, electrolytes, and liver enzymes. Also, a total body computed tomography (CT) was ordered to rule out head injury, intracranial bleed, and intra-abdominal bleed.

**Fig. 1:**
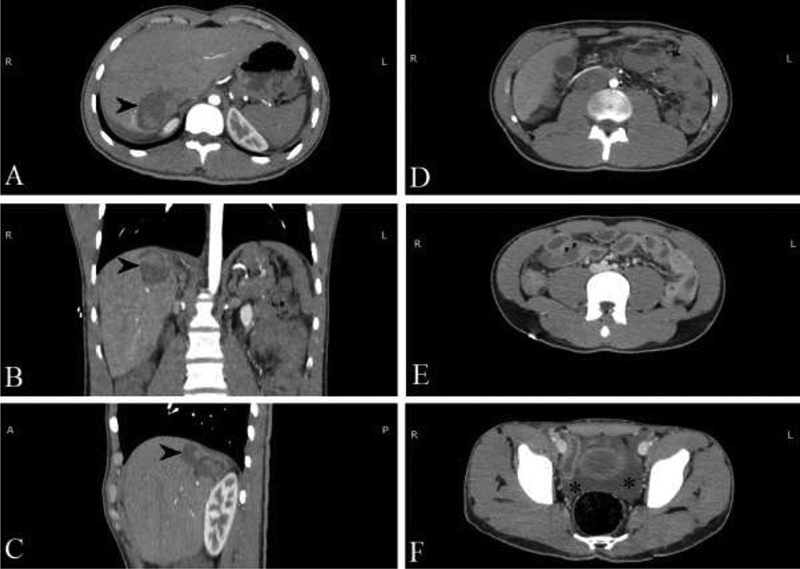
Computed tomography (CT) scan of the abdomen and pelvis + intravenous (IV) contrast. ( **A-C** ) Arrowhead points to a heterogeneous liver lesion. ( **D-E** ) Thickening of the gallbladder and small bowel wall. ( **F** ) Fluid collection in the pelvis marked by asterisk.

His laboratories were significant for: hemoglobin (Hb) 20 g/dL, white blood cell (WBC) 8800 m/mm ^3^ with N 35% and L 60%; everything else was within normal range. CT of the brain, neck, and chest was also normal. CT of the abdomen and pelvis showed ( Fig. 1 ) a heterogeneous liver lesion in segment VII, measuring 60 mm along its largest axis. This liver lesion was showed a rupture/discontinuity in the liver capsule but with no active extravasation of IV contrast, so active bleeding was ruled out. CT also showed thickening of the gallbladder wall and small bowel wall. In addition, we noted minimal peritoneal effusion in the perihepatic region and free fluid in the pelvis.

After the CT findings, we ordered a bedside ultrasound (US) to further delineate the liver lesion, and the radiologist raised suspicion of a ruptured liver cyst. Meanwhile, the patient became unstable. His temperature spiked to 39 °C, his systolic blood pressure (SBP) dropped to 80 mm Hg, his heart rate increased to 120 BPM, and his saturation dropped to 92%. Moreover, his abdomen became rigid. Hb was repeated and result was 17 g/dL.

Seeing that the patient was in shock and rapidly deteriorating, we decided to do an exploratory laparotomy to rule out any intra-abdominal bleeding or life-threatening pathology. Consent was taken, and we transferred the patient urgently to the operative theater.

**Fig. 2:**
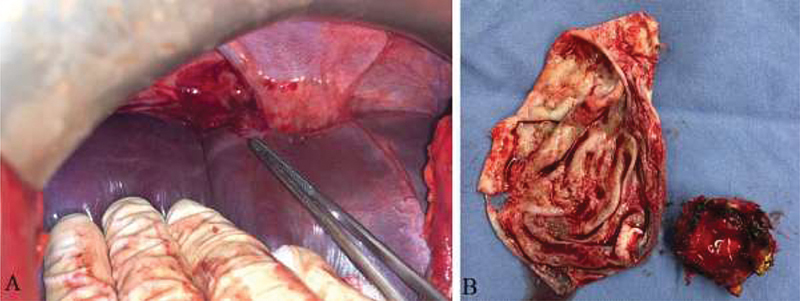
Intraoperative discovery. ( **A** ) Intraoperative view of protruding dome pointed by an instrument. ( **B** ) Removed hydatid cyst membrane (left) and resected protruding dome (right).

Under general anesthesia and with the patient in the supine position, we did a midline laparotomy incision. Exploration showed no signs of blood, active bleeding, or any gastrointestinal (GI) fluid leakage. Nevertheless, we found an abundant quantity of turbid fluid, mainly near the right subdiaphragmatic area. Suction and copious lavage were done using normal saline. Upon a closer look, a protruding dome in the liver parenchyma was noticed, around segment VII ( Fig. 2A ). It became clear that the patient's fall led to the rupture of a liver cyst. We excised the dome, which exposed a cavity containing a white membrane consistent with a hydatid cyst ( Fig. 2B ). We continued lavage with hypertonic saline 3%, and the patient stabilized progressively. We inset two drains, one inside the liver cavity, and the other in the right subdiaphragmatic area.

The patient tolerated well the procedure and was transferred to the intensive care unit (ICU) for surveillance. Echinococcal serology taken came back positive, and albendazole 100 mg (1 tab once daily) was started. Our patient improved over the next 3 days and was transferred to the regular floor. He was soon started on normal diet. Drains' output showed no bile leak, and the patient was discharged 1 week postop after removing the drains. He was given a prescription for albendazole to be taken over the following 6 months. The patient came back 10 days later for suture removal. His wound was clean, and he was asymptomatic. He was referred to an infectious disease specialist for follow-up.

## Discussion

The rupture of a liver hydatid cyst into the peritoneal cavity is very rare and ranges from 1% to 16% of patients with hydatid disease. 4 Several cases were found in the literature about hydatid cyst rupture into the peritoneal cavity. Most of them describe spontaneous rupture and only a handful are caused by traumatic situations. Nevertheless, trauma was reported as the most common cause of rupture. 5 Risk factors were found to be young age, superficial cysts, and large cysts (> 10 cm). Older patients with large and multiple cysts are mentioned to be at risk of spontaneous intrabiliary rupture. 5 The patient usually presents posttrauma with several complaints and injuries.

Signs and symptoms almost always include acute abdominal pain and peritoneal signs such as guarding and rebound tenderness. High fever might be present. Patients may also exhibit urticaria, macular eruptions, and in 25% of cases signs of anaphylaxis are found. In some cases, patients had a history of vague abdominal pain prior to the incident. 6 Surgeons and emergency department physicians in endemic areas should keep the diagnosis in mind when dealing with acute abdomen. In addition, as most people contract this parasite at a young age, doctors treating immigrants from endemic areas should keep the diagnosis in mind.

The screening tool for hydatid cysts is mainly the US. In cases of hydatid cyst perforation, US shows intra-abdominal fluid. It might also demonstrate intraperitoneal cysts. Nevertheless, CT scan claims its role in urgent presentations and allows for a multiorgan screening. Both modalities should be able to display heterogeneous cavity or cystic structures in the liver. 6 7 In addition, von Sinner indicated the “snake sign” and “spin sign” as particular for the diagnosis of hydatid cysts. 8 Magnetic resonance imaging (MRI) has no place in emergencies, but it is important to note that it is superior to CT and US in identifying biliary fistulae. 3 Laboratory findings are nonspecific but leukocytosis is common. Liver enzyme can be normal or disturbed. Leukopenia and thrombocytopenia can also be present. 4

When the diagnosis is made, medical treatment for the anaphylaxis should be administered promptly. Moreover, urgent surgical management should be undertaken. The main goals of surgery are to eliminate any disseminated disease, prevent complications, and minimize morbidity, mortality, and recurrence rate. 9 As such patients are highly unstable and cannot tolerate radical and morbid procedures, the choice of surgery is based on the fastest and less morbid method. For this reason, open conservative surgery (partial pericystectomy) is the gold standard. 7 9 10 The residual cavity could be left open, or further steps could be taken such as marsupialization, omentoplasty, or temporary drainage. Omentoplasty has been shown to be the most superior postconservative surgery. 11 It is very important to perform copious lavage of the peritoneal cavity with scolicidal agents to decrease the rate of peritoneal seeding and recurrence. 5 No clear consensus is available on the treatment of additional incidentally found cysts during surgery or on preoperative imaging. We suggest dealing with the extra cyst (s) during the same surgery, only if the patient is stable enough, and if the cysts require surgical treatment and cannot be managed by medical or percutaneous treatments.

Postoperative complications can be numerous. Early complications include pulmonary complications, wound infection/collection, intra-abdominal collection/abscess, biliary fistulae, end-organ damage due to shock, and even death. 5 For these reasons, we suggest that the patient should be monitored closely for the first 48 hours and given vasopressors when needed. Moreover, we highly recommend the placement of a drain, as it would give an early indication of a biliary fistula. Late postoperative complications include incisional hernia, recurrence and peritoneal hydatidosis, resulting from the disseminated scolices that grow in the peritoneum. 5

Surgery alone is not curative, and complicated hydatid cysts have a very high rate of recurrence. 11 For this reason, adjunctive medical treatment is recommended for at least 3 to 6 months, especially since traumatic cyst rupture requires emergency surgery, and patients fail to receive medical treatment before surgery. Therefore, medical treatment may be started as early as possible in the postoperative phase. Albendazole (10–15 mg/kg/day) is the drug used most commonly, as it has a superior absorption in the GI tract. 12 It is given as a 1-month course every 2 weeks for the desired period.

Patient follow-up is crucial for early detection of recurrence. Complicated cases, such as cyst rupture, are followed with ultrasonographic examination and indirect hemagglutination test every 3 months for the first year, then every 6 months for the following year, and annually up to 10 years. 6

## Conclusion

Complications of hydatid disease are serious and life-threatening. Rupture of cysts is rare but fatal if not treated in time. Workers in endemic areas should keep this diagnosis in mind while examining patients with acute abdomen. Management and treatment rely on urgent surgery. Follow-up is important as high rates of recurrence are expected.
